# Association between single nucleotide polymorphisms of TPH1 and TPH2 genes, and depressive disorders

**DOI:** 10.1111/jcmm.13459

**Published:** 2018-01-05

**Authors:** Paulina Wigner, Piotr Czarny, Ewelina Synowiec, Michał Bijak, Katarzyna Białek, Monika Talarowska, Piotr Galecki, Janusz Szemraj, Tomasz Sliwinski

**Affiliations:** ^1^ Laboratory of Medical Genetics Department of Molecular Genetics Faculty of Biology and Environmental Protection University of Lodz Lodz Poland; ^2^ Department of Medical Biochemistry Medical University of Lodz Lodz Poland; ^3^ Department of General Biochemistry Faculty of Biology and Environmental Protection University of Lodz Lodz Poland; ^4^ Department of Adult Psychiatry Medical University of Lodz Lodz Poland

**Keywords:** depression, tryptophan catabolites pathways, tryptophan hydroxylase, single nucleotide polymorphism

## Abstract

Tryptophan catabolites pathway disorders are observed in patients with depression. Moreover, single nucleotide polymorphisms of tryptophan hydroxylase genes may modulate the risk of depression occurrence. The objective of our study was to confirm the association between the presence of polymorphic variants of TPH1 and TPH2 genes, and the development of depressive disorders. Six polymorphisms were selected: c.804‐7C>A (rs10488682), c.‐1668T>A (rs623580), c.803+221C>A (rs1800532), c.‐173A>T (rs1799913)—*TPH1*, c.‐1449C>A (rs7963803), and c.‐844G>T (rs4570625)—*TPH2*. A total of 510 DNA samples (230 controls and 280 patients) were genotyped using TaqMan probes. Among the studied polymoorphisms, the G/G genotype and G allele of c.804‐7C>A—*TPH1*, the T/T homozygote of c.803+221C>A—*TPH1*, the A/A genotype and A allele of c.1668T>A—*TPH1*, the G/G homozygote and G allele of c.‐844G>T—*TPH2*, and the C/A heterozygote and A allele of c.‐1449C>A—*TPH2* were associated with the occurrence of depression. However, the T/T homozygote of c.‐1668T>A—*TPH1*, the G/T heterozygote and T allele of c.‐844G>T—*TPH2*, and the C/C homozygote and C allele of c.‐1449C>A—*TPH2* decreased the risk of development of depressive disorders**.** Each of the studied polymorphisms modulated the risk of depression for selected genotypes and alleles. These results support the hypothesis regarding the involvement of the pathway in the pathogenesis of depression.

## Introduction

Although the pathogenesis of depression (depressive disorder—DD) is not fully understood, studies suggest that disturbances in the TRYCATs pathway may play a key role in the development of this disease. A reduced level of tryptophan in plasma may lead to mood disorders in patients 1, 2. Moreover, increased plasma levels of harmful tryptophan metabolites—*i.e*. kynurenine, xanthurenic acid and quinolinic acid—were found in depressed patients 1, 2. Quinolinic acid may cause the destruction of postsynaptic structures and neurons *via* apoptosis of hippocampal cells and selective necrosis of granular cells. Additionally, it reduces the levels of dopamine, choline and γ‐aminobutyric acid (GABA) 3, 4, 5. On the other hand, studies showed that some tryptophan metabolites, for example kynurenic acid, may exhibit neuroprotective and antidepressant properties 2.

Recent findings have revealed that the patients with DD are characterized by greater activity of 2,3‐dioxygenase tryptophan (TDO) and 2,3‐dioxygenase indoleamine (IDO) as compared to healthy volunteers. Both are rate‐limiting enzymes in tryptophan metabolism 6. IDO/TDO converts tryptophan into kynurenine, which may be later metabolized into neurotoxic compounds, such as quinolinic acid. As a result, depressed patients are characterized by an increased kynurenine/tryptophan ratio and decreased serotonin/tryptophan ratio 1, 2.

Additionally, toxic TRYCATs can bring about increased production of reactive oxygen species (ROS). For example, 3‐hydroxykynurenine can induce neuronal apoptosis *via* overproduction of ROS 7. Moreover, kynurenine may penetrate the blood–brain barrier and exhibits toxic effects in the brain. As a consequence, it can lead to spreading cortical and subcortical atrophy 8.

Decreased levels of serotonin (5‐TH) or its receptors are also associated with depressed mood 9. This neurotransmitter is synthesized by tryptophan hydroxylase (TPH) 10. TPH is an enzyme which is involved in the initial and rate‐limiting step in the synthesis of serotonin and melatonin. It is responsible for the addition of a hydroxyl group to tryptophan and for the creation of amino acid 5‐hydroxytryptophan. There are two distinct genes encoding TPH in humans*—TPH1* and *TPH2*. Studies show that different expression levels of TPH genes may be related to aggression, schizophrenia, alcoholism, drug abuse, suicidality and depression 11, 12, 13, 14, 15. Placidi *et al*. 16 demonstrated that a low level of 5‐hydroxyindoleacetic acid (5‐HIAA) (the main metabolite of serotonin) in cerebrospinal fluid may be associated with suicidal attempts in DD. Moreover, a recent study has suggested that TPH2 expression in the midbrain is implicated in the antidepressant action of selective serotonin reuptake inhibitors (SSRI) 17. TPH2 may be a good candidate for a biomarker in pharmacogenetic studies of SSRI efficacy. Low levels of melatonin, which is also created in the TRYCATs pathway, are observed in patients suffering from Alzheimer's disease, carcinoma, anorexia and depression 18. About 80% of depressed patients exhibit different sleep disturbances. Persistent or worsening insomnia may contribute to the risk of depression recurrence and may increase its severity. In addition, severe insomnia occurs more frequently in the patients with depressive suicidal attempts than in the patients without such attempts 19. Therefore, in this article, we have decided to examine the relationship between six SNPs in the following genes responsible for encoding key TRYCAT enzymes: *TPH1* and *TPH2*.

## Materials and methods

### Volunteers

The study was carried out on a group of 280 patients suffering from DD, hospitalized at the Department of Adult Psychiatry of the Medical University of Lodz (Poland), and 230 healthy volunteers, randomly selected without replacement sampling. The volunteers taking part in the experiment were native Poles from central Poland (not related). The characteristics of the patients are presented in Table 1. The inclusion criteria were based on those outlined in ICD‐10 and APA (F32.0‐7.32.2, F330‐F33.8) 20, 21. The exclusion criteria included the presence of axis I and axis II disorders, other than DD, severe and chronic somatic diseases, injuries of the central nervous system, inflammatory or autoimmune disorders, and unwillingness to give informed consent. Additionally, volunteers with familial prevalence of mental disorders, other than recurrent depressive disorders, were excluded from the examined group. Medical history for all cases was obtained in accordance with the Standardized Composite International Diagnostic Interview (CIDI) prior to the start of the experiment 22. The 21‐item Hamilton Depression Rating Scale (HDRS‐21) was used to evaluate and classify depression severity 23. The scores presented in a study conducted by Demyttenaere and De Fruyt 24 were used in the measurements of intensity levels of DD symptoms. Each patient was examined by the same psychiatrist (CIDI and HDRS); psychiatric evaluation was performed before the patient was enrolled to take part in the study. Participation in the study was voluntary, and the volunteers were informed of the purpose, assured of the voluntary character of the experiment, and guaranteed that their personal data would be kept in secret before deciding to participate in the study. According to the protocol approved by the Bioethics Committee of the Medical University of Lodz (no. RNN/70/14/KE), all the volunteers consented to participate in the study.

**Table 1:** Characteristics of the investigated controls and patients

Characteristics	Controls (*n* = 230)	Patients (*n* = 280)
Sex (male/female)	114/116	148/132
Age (mean ± S.D.)	53.19 ± 12.61	49.53 ± 10.175
Age of onset (mean ± S.D.)	–	36.64 ± 10.89
HDRS‐21 (mean ± S.D.)	–	23.50 ± 6.14

### Selection of SNPs

The public domain of the database for SNPs of the National Center for Biotechnology Information (NCBI dbSNP), available at http://www.ncbi.nlm.nih.gov/snp (Bethesda, Montgomery County, MD, USA), was used to choose the studied polymorphisms. The selection criteria regarding SNPs were as follows: Their minor allele frequency had to be larger than 0.05 (submitter population ID: HapMap‐CEU), and they had to be localized either in the coding or regulatory region of the genes (Table 2).

**Table 2:** Characteristics of studied polymorphisms

Gene	rs number	Polymorphism	Localization
*TPH1*	rs1799913	c.804‐7C>A	near gene 5′
	rs623580	c.‐1668T>A	
	rs1800532	c.803+221C>A	Intron
	rs10488682	c.‐173A>T	
*TPH2*	rs7963803	c.‐1449C>A	near gene 5′
	rs4570625	c.‐844G>T	

### DNA extraction

Genomic DNA was isolated from venous blood according to the Blood Mini Kit protocol (A&A Biotechnology, Gdynia, Poland). Blood samples were collected from the patients suffering from DD before commencement of the antidepressant therapy. The purity of the DNA samples was measured spectrophotometrically by calculating the ratio between absorbance at 260 and 280 nm; after that, the samples were stored at −20°C.

### Genotyping

The chosen SNPs were genotyped using the TaqMans SNP Genotyping Assay (Thermo Fisher Scientific, Waltham, MA, USA) and 2× Master Mix Takyon for Probe Assay—No ROX (Eurogentec, Liège, Belgium). Reactions were performed according to the manufacturers’ instructions and recommendations. Real‐time PCRs were carried out in the Bio‐Rad CFX96 Real‐Time PCR Detection System and analysed in the CFX Manager Software (Bio‐Rad Laboratories Inc., Hercules, CA, USA).

### Statistical analysis

A statistical analysis of data was performed in Statistica 12 (Statsoft, Tulsa, OK, USA) and SigmaPlot 11.0 (Systat Software Inc., San Jose, CA, USA). An unconditional multiple logistic regression model was used to calculate the association between case/control and each polymorphism. The results are shown as odds ratios (ORs) with 95% confidence interval (95% CI). Additionally, the OR was adjusted for gender, as women are exposed to doubled risk of depression development in comparison to men 25. The data presenting the results from the distribution of genotypes in terms of the age of depression episode onset are shown as mean ± S.D. Distribution normality was examined using the Shapiro–Wilk test, and then, significance of the difference between studied values was determined based on the Mann–Whitney test or Student's *t*‐test.

## Results

### SNPs of the genes encoding TRYCATs enzymes (TPH1 and TPH2) as the risk of depressive disorders

The distribution of genotypes and alleles, as shown in Table 3, was in agreement with the Hardy–Weinberg equilibrium. Among the studied SNPs, the C/C genotype and the C allele of c.804‐7C>A*—TPH1* (rs1799913), the homozygote A/A of c.803+221C>A*—TPH1* (rs1800532), the T/T genotype and T allele of c.‐173A> T*—TPH1* (rs10488682) were significantly associated with an increased risk of DD. The T/T genotype of c.‐1668T>A*—TPH1* (rs623580) was negatively correlated with depression, while genotype A/A and allele A of the same SNP were positively correlated with the disease. In addition, the G/G genotype and G allele of c.‐844G>T*—TPH2* (rs4570625) were positively correlated with depression, whereas the G/T heterozygote and T allele of the same SNP were negatively correlated with the disease. The C/C genotype and C allele of c.‐1449C>A*—TPH2* (rs7963803) were negatively correlated with DD, while heterozygote C/A and allele A of the same SNP were positively correlated with the disease.

**Table 3:** Distribution of genotypes and alleles of c.804‐7C>A, c.‐1668T>A, c.803+221C>A, c.‐173A>T, c.‐1449C>A and c.‐844G>T and the risk of DD

Genotype/Allele	Control (*n* = 230)		Depression (*n* = 280)		Crude OR (95% CI)^a^	*P*	Adjusted OR (95% CI)^a^	*P*
	Number	Frequency	Number	Frequency				
c.804‐7C>A ‐ *TPH1* (rs1799913)								
** C/C**	**65**	**0.283**	**100**	**0.357**	**1.473 (1.013–2.141)**	**0.042**	**1.413 (0.969–2.060)**	**0.073**
C/A	118	0.513	127	0.454	0.865 (0.613**–**1.222)	0.411	0.787 (0.555**–**1.117)	0.180
A/A	47	0.204	53	0.189	0.964 (0.623**–**1.492)	0.868	0.908 (0.586**–**1.408)	0.666
χ^2^ = 2.131; *P = *0.345								
** C**	**248**	**0.539**	**327**	**0.584**	**1.288 (1.012–1.639)**	**0.040**	**1.196 (0.935–1.531)**	**0.154**
A	212	0.461	233	0.416	0.911 (0.715**–**1.160)	0.448	0.836 (0.653**–**1.070)	0.154
c.803+221C>A ‐ *TPH1* (rs1800532)								
C/C	66	0.287	93	0.331	1.319 (0.905**–**1.922)	0.150	1.212 (0.831**–**1.769)	0.318
C/A	151	0.657	158	0.564	0.772 (0.543**–**1.098)	0.149	**0.676 (0.471–0.969)**	**0.033**
** A/A**	**11**	**0.048**	**29**	**0.104**	**2.416 (1.180–4.947)**	**0.016**	**2.308 (1.126–4.730)**	**0.022**
χ^2^ = 0.249; *P = *0.883								
C	283	0.615	344	0.614	1.160 (0.872**–**1.544)	0.307	0.970 (0.719**–**1.309)	0.843
A	173	0.376	216	0.386	1.170 (0.871**–**1.571)	0.298	1.058 (0.784**–**1.429)	0.712
c.‐173A>T ‐ *TPH1* (rs10488682)								
**T/T**	**119**	**0.517**	**167**	**0.596**	**1.515 (1.070–2.145)**	**0.019**	1.377 (0.968**–**1,958)	0.075
A/T	99	0.430	98	0.35	0.772 (0.541**–**1.102)	0.154	0.713 (0.498**–**1.020)	0.064
A/A	12	0.052	15	0.054	1.080 (0.495**–**2.355)	0.846	1.032 (0.473**–**2.252)	0.937
χ^2 ^= 2.221; *P = *0.329								
**T**	**337**	**0.733**	**432**	**0.771**	**1.444 (1.095–1.904)**	**0.009**	**1.242 (0.927–1.664)**	**0.147**
A	123	0.267	128	0.229	0.860 (0.644**–**1.149)	0.309	0.805 (0.601**–**1.079)	0.147
c.‐1668T>A ‐ *TPH1* (rs623580)								
**T/T**	**121**	**0.267**	**116**	**0.414**	**0.701 (0.496–0.992)**	**0.045**	**0.638 (0.449–0.908)**	**0.012**
T/A	95	0.733	132	0.471	1.371 (0.967**–**1.943)	0.077	1.264 (0.888**–**1.798)	0.193
**A/A**	**14**	**0.061**	**32**	**0.114**	**2.092 (1.089–4.021)**	**0.027**	**1.996 (1.037–3.839)**	**0.038**
χ^2^ = 8.468; ***P = *** **0.014**								
T	337	0.413	364	0.65	0.800 (0.617**–**1.039)	0.094	**0.667 (0.505–0.881)**	**0.004**
**A**	**123**	**0.526**	**196**	**0.35**	**1.598 (1.212–2.107)**	**<0.001**	**1.500 (1.136–1.981)**	**0.004**
c.‐844G>T ‐ *TPH2* (rs4570625)								
**G/G**	**48**	**0.209**	**167**	**0.596**	**5.942 (3.999–8.831)**	**<0.001**	**5.647 (3.790–8.413)**	**<0.001**
**G/T**	**179**	**0.778**	**111**	**0.396**	**0.223 (0.153–0.324)**	**<0.001**	**0.186 (0.125–0.275)**	**<0.001**
T/T	3	0.013	2	0.007	0.571 (0.0946**–**3.444)	0.541	0.546 (0.0905**–**3.299)	0.510
χ^2^ = 78.662; ***P < *** **0.001**								
** G**	**275**	**0.598**	**445**	**0.794**	**5.496 (3.764–8.027)**	**<0.001**	**5.213 (3.632–7.695)**	**<0.001**
** T**	**185**	**0.402**	**115**	**0.205**	**0.230 (0.159–0.333)**	**<0.001**	**0.192 (0.130–0.283)**	**<0.001**
c.‐1449C>A ‐ *TPH2* (rs7963803)								
** C/C**	**114**	**0.496**	**96**	**0.343**	**0.581 (0.408–0.828)**	**0.003**	**0.529 (0.370–0.759)**	**<0.001**
** C/A**	**106**	**0.461**	**178**	**0.636**	**2.223 (1.563–3.160)**	**<0.001**	**2.054 (1.438–2.934)**	**<0.001**
A/A	10	0.043	6	0.021	0.506 (0.181**–**1.413)	0.193	0.478 (0.171**–**1.338)	0.160
χ^2 ^= 7.447; ***P = *** **0.024**								
** C**	**334**	**0.726**	**370**	**0.661**	**0.823 (0.609–1.111)**	**0.203**	**0.641 (0.463–0.886)**	**0.007**
** A**	**126**	**0.274**	**190**	**0.339**	**1.694 (1.228–2.335)**	**0.001**	**1.561 (1.128–2.159)**	**0.007**

a OR adjusted for sex.

*P < *0.05 along with corresponding ORs are in bold.

### SNPs of the genes encoding TRYCATs enzymes and the age of the first episode of depression, and the severity classification on the Hamilton Depression Rating Scale

**Figure 1:**
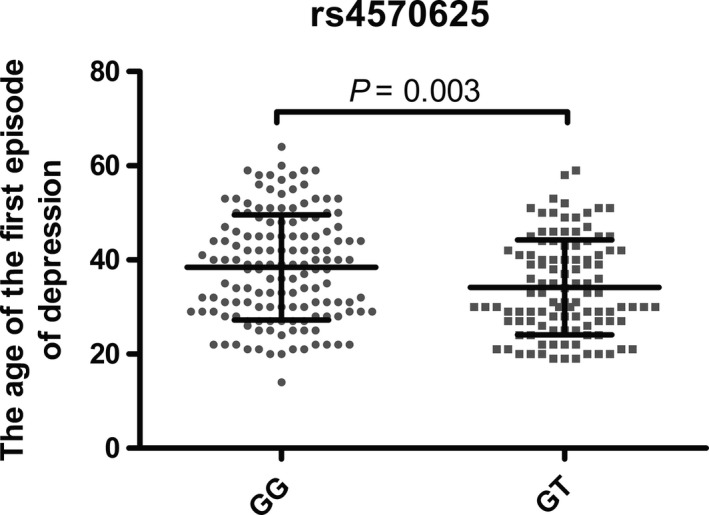
Distribution of single nucleotide polymorphisms of genes encoding TPH2 and the age of the first episode of depression. Horizontal lines denote the average, while whiskers show the S.D. The distribution of the T/T genotype is not shown because this group included only two patients.

We only found one difference in the age distribution of the first depressive episode between the G/G and G/T genotypes of the c.‐844G>T*—TPH2* (rs4570625) polymorphism (Figs 1 and S1). We did not find any significant differences in the distribution of genotypes and the severity classification on the Hamilton Depression Rating Scale (data unpublished).

### Gene–gene interactions and the risk of depression

We also studied whether the combined genotypes of the studied polymorphisms are associated with the occurrence of depression, and the results are presented in Table 4. We observed that the C/C‐G/G, C/A‐G/G, A/A‐G/G genotypes of c.804‐7C>A*—TPH1* and c.‐844G>T*—TPH2* (rs1799913 *versus* rs4570625) were associated with an increased risk of DD occurrence, while the C/A‐G/T, A/A‐G/T genotypes of the same polymorphism combination reduced this risk. We also found that the C/A‐C/C combined genotype of the c.804‐7C>A*—TPH1* and c.‐1449C>A*—TPH2* (rs1799913 *versus* rs7963803) was related to a decreased risk of the disease, but the C/A‐C/A combined genotype of the same polymorphism may increase this risk.

**Table 4:** Gene–gene interactions of studied polymorphisms and the risk of DD

Combined genotype	Control (*n* = 230)		Depression (*n* = 280)		Crude OR (95% CI)	*P*	Adjusted OR (95% CI)^a^	*P*
	Number	Frequency	Number	Frequency				
c.804‐7C>A*—TPH1* (rs1799913) and c.‐844G>T*—TPH2* (rs4570625)								
** C/C‐G/G**	**17**	**0.074**	**60**	**0.214**	**3.594 (2.033–6.353)**	**<0.001**	**3.428 (1.936–6.070)**	**<0.001**
C/C‐G/T	48	0.209	39	0.139	0.651 (0.410**–**1.034)	0.069	**2.181 (1.089–4.366)**	**0.028**
C/C‐T/T	0	0	1	0.004	**–**	0.986	a	0.986
** C/A‐G/G**	**25**	**0.109**	**74**	**0.264**	**3.104 (1.898–5.076)**	**<0.001**	**2.947 (1.1800–4.824)**	**<0.001**
** C/A‐G/T**	**92**	**0.4**	**52**	**0.186**	**0.369 (0.248–0.550)**	**<0.001**	**0.342 (0.229–0.511)**	**<0.001**
C/A‐T/T	1	0.004	1	0.004	0.860 (0.0535**–**13.827)	0.915	0.821 (0.0511**–**13.199)	0.889
** A/A‐G/G**	**6**	**0.026**	**33**	**0.118**	**5.233 (2.153–12.717)**	**<0.001**	**4.988 (2.051–12.127)**	**<0.001**
** A/A‐G/T**	**39**	**0.170**	**20**	**0.071**	**0.398 (0.225–0.704)**	**0.002**	**0.377 (0.213–0.667)**	**<0.001**
A/A‐T/T	2	0.009	0	0	**–**	**–**	**–**	**–**
c.804‐7C>A*—TPH1* (rs1799913) and c.‐1449C>A*—TPH2* (rs7963803)								
C/C‐C/C	30	0.130	41	0.146	1.207 (0.727**–**2.001)	0.467	1.143 (0.688**–**1.899)	0.605
C/C‐C/A	32	0.139	55	0.196	1.597 (0.993**–**2.566)	0.053	1.513 (0.940**–**2.434)	0.088
C/C‐A/A	3	0.013	4	0.014	1.150 (0.255**–**5.189)	0.856	1.096 (0.243**–**4.949)	0.905
** C/A‐C/C**	**62**	**0.269**	**39**	**0.139**	**0.467 (0.299–0.729)**	**<0.001**	**0.438 (0.280–0.685)**	**<0.001**
** C/A‐C/A**	**52**	**0.226**	**86**	**0.307**	**1.611 (1.082**–**2.400)**	**0.019**	**1.517 (1.017**–**2.264)**	**0.041**
C/A‐A/A	4	0.017	2	0.007	0.426 (0.0774–2.348)	0.327	0.406 (0.0733–2.246)	0.302
A/A‐C/C	22	0.096	16	0.057	0.603 (0.309–1.177)	0.138	0.571 (0.292–1.119)	0.102
A/A‐C/A	22	0.096	37	0.132	1.516 (0.867–2.649)	0.144	1.440 (0.822–2.522)	0.202
A/A‐A/A	3	0.013	0	0	**–**	**–**	**–**	**–**
c.803+221C>A*—TPH1* (rs1800532) and c.‐844G>T*—TPH2* (rs4570625)								
** C/C‐G/G**	**17**	**0.074**	**55**	**0.196**	**3.221 (1.813**–**5.721)**	**<0.001**	**3.063 (1.723**–**5.446)**	**<0.001**
** C/C‐G/T**	**50**	**0.217**	**37**	**0.132**	**0.582 (0.365**–**0.926)**	**0.022**	**0.548 (0.343**–**0.874)**	**0.012**
C/C‐T/T	0	0	1	0.004	**–**	**–**	**–**	**–**
** C/A‐G/G**	**26**	**0.113**	**93**	**0.332**	**4.113 (2.553**–**6.626)**	**<0.001**	**3.903 (2.420**–**6.294)**	**<0.001**
** C/A‐G/T**	**125**	**0.543**	**64**	**0.229**	**0.275 (0.189**–**0.401)**	**<0.001**	**0.248 (0.169**–**0.363)**	**<0.001**
C/A‐T/T	1	0.004	1	0.004	0.860 (0.0535–13.827)	0.915	0.821 (0.0511–13.199)	0.889
** A/A‐G/G**	**5**	**0.022**	**19**	**0.068**	**3.436 (1.263**–**9.347)**	**0.016**	**3.284 (1.205**–**8.950)**	**0.020**
A/A‐G/T	4	0.017	10	0.034	2.194 (0.679–7.089)	0.189	2.096 (0.648–6.778)	0.216
A/A‐T/T	2	0.009	0	0	**–**	**–**	**–**	**–**
c.803+221C>A*—TPH1* (rs1800532) and c.‐1449C>A*—TPH2* (rs7963803)								
C/C‐C/C	33	0.143	38	0.136	0.990 (0.599–1.635)	0.968	0.937 (0.566–1.590)	0.799
C/C‐C/A	32	0.139	53	0.189	1.525 (0.946–2.458)	0.083	1.445 (0.895–2.331)	0.132
C/C‐A/A	2	0.009	2	0.007	0.860 (0.120–6.150)	0.880	0.820 (0.115–5.870)	0.844
**C/A‐C/C**	**77**	**0.335**	**50**	**0.179**	**0.463 (0.308**–**0.697)**	**<0.001**	**0.430 (0.285**–**0.650)**	**<0.001**
**C/A‐C/A**	**67**	**0.291**	**104**	**0.371**	**1.535 (1.058**–**2.226)**	**0.024**	1.438 (0.989–2.089)	0.057
C/A‐A/A	8	0.035	4	0.014	0.422 (0.126–1.420)	0.163	0.402 (0.119–1.354)	0.141
A/A‐C/C	4	0.017	8	0.029	1.743 (0.518–5.860)	0.369	1.662 (0.494–5.592)	0.412
**A/A‐C/A**	**7**	**0.030**	**21**	**0.075**	**2.710 ()1.132**–**6.492**	**0.025**	**2.584 (1.078**–**6.196)**	**0.033**
A/A‐A/A	0	0	0	0	**–**	**–**	**–**	**–**
c.‐173A>T*—TPH1* (rs10488682) and c.‐844G>T*—TPH2* (rs4570625)								
**T/T‐G/G**	**22**	**0.096**	**101**	**0.361**	**5.617 (3.401**–**9.276)**	**<0.001**	**5.337 (3.228**–**8.822)**	**<0.001**
**T/T‐G/T**	**96**	**0.417**	**65**	**0.232**	**0.457 (0.313**–**0.667)**	**<0.001**	**0.422 (0.288**–**0.618)**	**<0.001**
T/T‐T/T	1	0.004	1	0.004	0.860 (0.0535–13.827)	0.915	0.821 (0.0511–13.199)	0.889
**A/T‐G/G**	**25**	**0.109**	**58**	**0.207**	**2.257 (1.362**–**3.740)**	**0.002**	**2.143 (1.292**–**3.555)**	**0.003**
**A/T‐G/T**	**72**	**0.313**	**39**	**0.139**	**0.380 (0.245**–**0.588)**	**<0.001**	**0.355 (0.299**–**0.550)**	**<0.001**
A/T‐T/T	2	0.009	1	0.004	0.428 (0.0386–4.753)	0.490	0.408 (0.0367–4.529)	0.465
A/A‐G/G	1	0.004	8	0.029	7.059 (0.877–56.847)	0.066	6.746 (0.837–54.350)	0.073
A/A‐G/T	11	0.048	7	0.025	0.536 (0.205–1.405)	0.205	0.509 (0.194–1.336)	0.170
A/A‐T/T	0	0	0	0	**–**	**–**	**–**	**–**
c.‐173A>T*—TPH1* (rs10488682) and c.‐1449C>A*—TPH2* (rs7963803)								
T/T‐C/C	58	0.252	59	0.211	0.842 (0.558–1.272)	0.414	0.792 (0.523–1.198)	0.269
**T/T‐C/A**	**52**	**0.226**	**104**	**0.371**	**2.148 (1.453**–**3.176)**	**<0.001**	**2.023 (1.366**–**2.996)**	**<0.001**
T/T‐A/A	9	0.039	4	0.014	0.374 (0.114–1.229)	0.105	0.356 (0.108–1.172)	0.089
**A/T‐C/C**	**50**	**0.217**	**29**	**0.104**	**0.441 (0.269**–**0.724)**	**0.001**	**0.416 (0.253**–**0.683)**	**<0.001**
A/T‐C/A	48	0.209	67	0.239	1.265 (0.832–1.922)	0.272	1.192 (0.783–1.815)	0.412
A/T‐A/A	1	0.004	2	0.007	1.727 (0.156–19.160)	0.656	1.651 (0.149–18.346)	0.683
A/A‐C/C	6	0.026	8	0.0289	1.152 (0.394–3.368)	0.796	1.098 (0.375–3.210)	0.865
A/A‐C/A	6	0.026	7	0.025	1.004 (0.333–3.030)	0.994	0.956 (0.317–2.888)	0.937
A/A‐A/A	0	0	0	0	**–**	**–**	**–**	**–**
c.‐1668T>A*—TPH1* (rs623580) and c.‐844G>T*—TPH2* (rs4570625)								
**T/T‐G/G**	**23**	**0.1**	**72**	**0.257**	**3.281 (1.977**–**5.444)**	**<0.001**	**3.119 (1.877**–**5.182)**	**<0.001**
**T/T‐G/T**	**96**	**0.417**	**42**	**0.15**	**0.267 (0.176**–**0.405)**	**<0.001**	**0.246 (0.162**–**0.375)**	**<0.001**
T/T‐T/T	2	0.009	2	0.007	0.860 (0.120–6.150)	0.880	0.820 (0.115–5.870)	0.844
**T/A‐G/G**	**22**	**0.096**	**74**	**0.264**	**3.576 (2.142**–**5.970)**	**<0.001**	**3.398 (2.033**–**5.679)**	**<0.001**
**T/A‐G/T**	**72**	**0.313**	**58**	**0.207**	**0.613 (0.411**–**0.914)**	**0.016**	**0.573 (0.383**–**0.856)**	**0.007**
T/A‐T/T	1	0.004	0	0	**–**	0.986	**–**	0.985
**A/A‐G/G**	**3**	**0.013**	**21**	**0.075**	**6.432 (1.894**–**21.841)**	**0.003**	**6.145 (1.808**–**20.884)**	**0.004**
A/A‐G/T	11	0.048	11	0.039	0.855 (0.364–2.009)	0.719	0.814 (0.346–1.914)	0.638
A/A‐T/T	0	0	0	0	**–**	**–**	**–**	**–**
c.‐1668T>A*—TPH1* (rs623580) and c.‐1449C>A*—TPH2* (rs7963803)								
**T/T‐C/C**	**65**	**0.283**	**37**	**0.132**	**0.412 (0.263**–**0.645)**	**<0.001**	**0.386 (0.246**–**0.605)**	**<0.001**
T/T‐C/A	55	0.239	78	0.279	1.306 (0.877–1.945)	0.189	1.228 (0.823–1.833)	0.314
T/T‐A/A	1	0.004	1	0.004	0.860 (0.0535–13.827)	0.915	0.816 (0.0505–13.188)	0.886
T/A‐C/C	40	0.174	44	0.157	0.937 (0.587–1.496)	0.785	0.886 (0.554–1.417)	0.613
**T/A‐C/A**	**49**	**0.213**	**84**	**0.3**	**1.679 (1.120**–**2.517)**	**0.012**	**1.583 (1.055**–**2.376)**	**0.027**
T/A‐A/A	6	0.026	4	0.014	0.568 (0.158–2.036)	0.385	0.540 (0.150–1.948)	0.346
A/A‐C/C	9	0.039	15	0.054	1.459 (0.627–3.397)	0.381	1.390 (0.597–3.239)	0.445
**A/A‐C/A**	**2**	**0.009**	**16**	**0.057**	**7.242 (1.648**–**31.827)**	**0.009**	**6.913 (1.572**–**30.398)**	**0.011**
A/A‐A/A	3	0.013	1	0.004	0.284 (0.0294–2.752)	0.278	0.270 (0.0279–2.618)	0.259

a OR adjusted for sex.

*P < *0.05 along with corresponding ORs are in bold.

The C/C‐G/G, C/A‐G/G, A/A‐G/G combined genotype of c.803+221C>A*—TPH1* and c.‐844G>T*—TPH2* (rs180532 *versus* rs4570625) was linked with an increased risk of DD occurrence, while the C/C‐G/T, C/A‐G/T genotype of the same polymorphism combination decreased this risk. The C/A‐C/A and A/A‐C/A combined genotypes of the c.803+221C>A*—TPH1* and c.‐1449C>A*—TPH2* (rs1800532 *versus* rs7963803) may lead to a development of depression, and the C/A‐C/C combined genotype of the same SNPs combination reduce the risk of developing the disease.

The T/T‐G/G and T/A‐G/G combined genotypes of c.‐173A>T*—TPH1* and c.‐844G>T*—TPH2* (rs10488682 *versus* rs4570625) were associated with DD development, but the T/T‐G/T and the T/A‐G/T genotype of the same SNPs combination decreased the risk. Additionally, the increased risk of DD occurrence was associated with the T/T‐C/A combined genotype of c.‐173A>T*—TPH1* and c.‐1449C>A*—TPH2* (rs10488682 *versus* rs7963803); however, the T/A‐C/C genotype decreased the risk depression occurrence.

The T/T‐G/G, T/A‐G/G, A/A‐G/G combined genotypes of c.‐1668T>A*—TPH1* and c.‐844G>T*—TPH2* (rs623580 *versus* rs4570625) contributed to the development of DD, while T/T‐G/T and T/A‐G/G of the same SNP‐SNP combinations decreased this risk. In case of c.‐1668T>A*—TPH1* and c.‐1449C>A*—TPH2* (rs623580 *versus* rs7963803), combined T/A‐C/A and A/A‐C/A genotypes were associated with the occurrence of DD, while the T/T‐C/C genotype of the same polymorphism combination decreased this risk.

In summary, we found that the A/A‐G/G combined genotype of c.804‐7C>A*—TPH1* and c.‐844G>T*—TPH2* (rs1799913 and rs4570625) was associated with a five‐time higher risk of DD occurrence (*P < *0.001). In the case of c.803+221C>A*—TPH1* (rs1800532) and c.‐844G>T*—TPH2* (rs4570625), the C/A‐G/G combined genotypes were associated with the risk of depression higher by nearly four times (*P < *0.001), while the C/A‐G/T combined genotypes of the same polymorphism combination decreased this risk by more than three times (*P < *0.001). Moreover, the T/T‐G/G combined genotypes of c.‐173A>T*—TPH1* (rs10488682) and c.‐844G>T*—TPH2* (rs4570625) caused a five‐time greater risk among the Polish population (*P < *0.001). The risk of DD occurrence decreased by just four times in case of the T/T‐G/G combined genotypes of c.‐1668T>A*—TPH1* (rs623580) and c.‐844G>T*—TPH2* (rs4570625) (*P < *0.001), while the A/A‐G/G genotypes of the same gene–gene combination were linked to a six‐time higher risk (*P = *0.004). An increase by seven times of the risk of depression was confirmed for the A/A‐C/A combined genotypes of c.‐1668T>A*—TPH1* (rs623580) and c.‐1449C>A*—TPH2* (rs7963803) (*P = *0.011).

### Haplotypes and the risk of depression

The association between depression and haplotypes of the studied polymorphisms of the *TPH1* or *TPH2* genes was also assessed (Table 5.). The presence of CC and CA haplotypes of c.804‐7C>A*—TPH1* (rs1799913) and c.803+221C>A*—TPH1* (rs1800532) resulted in an increased risk of DD occurrence. We also noticed a link between the CA haplotype of c.804‐7C>A*—TPH1* (rs1799913) and c.‐1668T>A*—TPH1* (rs623580), and the increased rate of depression. In case of c.‐173A>T*—TPH1* (rs10488682) and c.803+221C>A*—TPH1* (rs1800532), the TA haplotype increased the risk of DD development, while the AA haplotype of the same SNPs combination reduced this risk. The TA, AC and AA haplotypes of c.‐1668T>A*—TPH1* (rs623580) and c.803+221C>A*—TPH1* (rs1800532) were responsible for an increased risk of depression development, while the TC haplotype of the same polymorphism combination decreased this risk. In case of c.‐173A>T (rs10488682) and c.‐1668T>A*—TPH1* (rs623580), the TA haplotype was associated with the occurrence of the disease. The CG and AG haplotypes of c.‐1449C>A*—TPH2* (rs7963803) and c.‐844G>T*—TPH2* (rs4570625) were connected with an increased risk of the disease, while the CT haplotype of the same SNPs combination increased the risk.

**Table 5:** Distribution of haplotypes of the studied polymorphisms of the *TPH1 or TPH2* genes and risk of the depression

Haplotype	Control (*n* = 230)		Depression (*n* = 280)		Crude OR (95% CI)	*P*
	Number	Frequency	Number	Frequency		
c.804‐7C>A*—TPH1* (rs1799913) and c.803+221C>A*—TPH1* (rs1800532)						
** CC**	**359**	**0.39**	**467**	**0.42**	**1.320 (1.101**–**1.581)**	**0.003**
** CA**	**137**	**0.15**	**187**	**0.17**	**1.283 (1.008**–**1.633)**	**0.043**
AC	213	0.23	221	0.20	0.918 (0.742–1.137)	0.433
AA	211	0.23	245	0.22	1.062 (0.861–1.311)	0.574
c.804‐7C>A*—TPH1* (rs1799913) and c.‐173A>T –TPH1 (rs10488682)						
CT	339	0.37	478	0.43	0.970 (0.807–1.1652)	0.743
CA	157	0.17	176	0.16	1.013 (0.800–1.284)	0.912
AT	335	0.36	386	0.34	1.063 (0.884–1.279)	0.651
AA	89	0.10	80	0.07	0.795 (0.579–1.090)	0.154
c.804‐7C>A*—TPH1* (rs1799913) and c.‐1668T>A ‐ TPH1 (rs623580)						
TT	353	0.38	384	0.34	0.838 (0.699–1.005)	0.056
** CA**	**143**	**0.16**	**270**	**0.24**	**1.726 (1.378**–**2.161)**	**<0.001**
AT	321	0.35	344	0.31	0.950 (0.787–1.146)	0.540
AA	103	0.11	122	0.11	1.078 ()0.815–1.424)	0.599
c.‐173A>T*—TPH1* (rs10488682) and c.803+221C>A*—TPH1* (rs1800532)						
TC	403	0.44	491	0.44	1.191 (0.996–1.424)	0.056
** TA**	**271**	**0.29**	**373**	**0.33**	**1.381 (1.141**–**1.671)**	**<0.001**
AC	169	0.18	197	0.188	1.060 (0.844–1.331)	0.500
** AA**	**77**	**0.08**	**59**	**0.05**	**0.672 (0.473**–**0.955)**	**0.027**
c.‐1668T>A*—TPH1* (rs623580) and c.803+221C>A*—TPH1* (rs1800532)						
** TC**	**413**	**0.45**	**393**	**0.35**	**0.770 (0.642**–**0.922)**	**0.005**
** TA**	**261**	**0.28**	**335**	**0.30**	**1.583 (1.308**–**1.916)**	**<0.001**
** AC**	**159**	**0.17**	**295**	**0.26**	**1.948 (1.566**–**2.422)**	**<0.001**
** AA**	**87**	**0.09**	**97**	**0.09**	**1.006 (0.742**–**1.364)**	**0.040**
c.‐173A>T (rs10488682) and c.‐1668T>A*—TPH1* (rs623580)						
TT	469	0.51	518	0.46	0.992 (0.830–1.186)	0.932
** TA**	**205**	**0.22**	**346**	**0.31**	**1.791 (1.463**–**2.192)**	**<0.001**
AT	205	0.22	210	0.19	0.904 (0.728–1.124)	0.364
AA	41	0.04	46	0.04	1.013 (0.658–1.558)	0.955
c.‐1449C>A*—TPH2* (rs7963803) and c.‐844G>T*—TPH2* (rs4570625)						
** CG**	**389**	**0.42**	**588**	**0.53**	**1.858 (1.551**–**2.225)**	**<0.001**
** CT**	**279**	**0.30**	**152**	**0.14**	**0.402 (0.322**–**0.503)**	**<0.001**
** AG**	**161**	**0.18**	**302**	**0.27**	**1.983 (1.596**–**2.463)**	**<0.001**
AT	91	0.10	78	0.07	0.754 (0.550–1.035)	0.081

*P < *0.05 along with corresponding ORs are in bold.

In summary, the haplotype analysis revealed that the AC haplotype of c.‐1668T>A*—TPH1* (rs623580) and c.803+221C>A*—TPH1* (rs1800532) nearly doubled the risk (*P < *0.001). In case of c.‐1449C>A*—TPH2* (rs7963803) and c.‐844G>T*—TPH2* (rs4570625), the CT haplotype increased risk of DD by almost two times (*P < *0.001), while the AG haplotype of the same combination decreased the risk almost twice (*P < *0.001).

## Discussion

A growing body of evidence and data suggests that when impaired the TRYCATs pathway may play an essential role in the development of depression 1, 2. As mentioned in the Introduction, these abnormalities may be linked with the irregular functioning of pathway enzymes, such as TPH. Two isoforms of TPH*—i.e. TPH1* and *TPH2*, can be found in humans and in other mammals. The human *TPH1* and *TPH2* are highly homologous proteins which exhibit 71% of amino acid identity. The human *TPH1* is located on chromosome 11p15.3‐14, comprises 11 exons and covers a region of 29 kb; the human TPH2 gene is located on chromosome 12q15, comprises 11 exons and covers a region of 97 kb 26. TPH1 and TPH2 are expressed in almost equal amounts in only a few regions of the brain such as the frontal cortex, thalamus, hippocampus, hypothalamus and amygdale, whereas TPH1 is also expressed in peripheral tissues such as the heart, lung, kidney, duodenum and the adrenal gland 27. TPH1 and TPH2 are essential enzymes for the correct metabolism of tryptophan. Both are also considered to be the rate‐limiting enzymes in serotonin biosynthesis 10. TPH converts L‐tryptophan into L‐5‐hydroxytryptophan (serotonin precursor) by means of adding an ‐HO group (hydroxylation) to position 5 of L‐tryptophan. Disturbances in their amount or activity may lead to deficiencies of neuroprotective compounds such as kynurenic acid and, consequently, to the occurrence of mood disorders. Polymorphic variants or altered expression levels of TPH genes may be related to depression, schizophrenia, alcoholism, drug abuse, aggression and suicidality 13, 14, 15, 28.

During this experiment, we genotyped six SNPs: four in *TPH1* and two in *TPH2*. We confirmed that the selected genotypes and alleles of four SNPs localized in *TPH1* modulated the risk of depression (Table 3). One of them, that is c.804‐7C>A*—TPH1* (rs1799913), is localized at intron 7 of the TPH1 gene and also at the polypyrimidine stretch immediately upstream of the 3′acceptor splice site. Although substitution of pyrimidine for purine in the polypyrimidine consensus sequence has been shown to decrease the fidelity of splicing, sequencing of TPH1 cDNA revealed no evidence of exon skipping or aberrant splicing 28. The studies showed that the c.804‐7C>A polymorphism was associated with 5‐HIAA concentrations in CSF 29. Moreover, the meta‐analysis confirmed that the link between TPH1 and bipolar disorders was not clear 30. Thus far, it has been confirmed that the polymorphism is associated with depression treatment through an assessment of the harm avoidance and novelty seeking 31. Andre *et al*. 31 found a greater effect of interactions between the CC genotype and remission status as compared to A‐allele carriers. Other studies demonstrated that the TPH1 polymorphism in the Swedish population may lead to unipolar disorders, suicidal behaviour and substance abuse 32. Gizatullin *et al*. 33 discovered that the c.804‐7C>A polymorphism was associated with depression in the Caucasian population of the North European descent. Our study, conducted among the Polish population, confirmed the results recorded by Gizatullin's team that selected genotypes and alleles of c.‐173A>T (rs1799913) could contribute to genetic predisposition for DD (Table 3). However, Gizatullin *et al*. analysed only haplotypes for six TPH1 SNPs, whereas our team showed also that the gene–gene combination of c.‐173A>T (rs1799913)*—TPH1* and c.‐844G>T (rs4570625)*—TPH2* or c.‐1449C>A (rs7963803)*—TPH2* may also modulate the risk of depression development. Moreover, haplotype analyses performed in both studies confirmed that the examined haplotypes may be associated with the occurrence of DD.

Another TPH1 polymorphism studied during this experiment is c.‐173A>T (rs10488682). The SNP is localized in the promoter region of TPH1 34 (Cote *et al*., 2002). It may decrease the activity of the promoter, affecting the transcription level of TPH1. TPH is an initial enzyme in the TRYCATs pathway, and its low expression may lead to stopping the pathway. The SNP was associated with the occurrence of adolescent idiopathic scoliosis. Patients with the A allele of this SNP are prone to be resistant to brace treatment 35. In turn, we confirmed that the G/G genotype and G allele of c.804‐7C>A*—TPH1* (rs10488682) may lead to the development of DD (Table 3).

The c.‐1668T>A SNP (rs623580) is localized in the exon 1c/intron 1 region, but the polymorphism is within the 5′UTR and therefore does not result in the substitution of amino acids 36. Studies involving the c.‐1668T>A polymorphism revealed the negative results linked with affective disorders and suicide‐related behaviour 37, 38. Ching‐Lopes *et al*., similarly to our team, discovered that the c.‐1668T>A polymorphism of TPH1 may increase the risk of depression 39, but an earlier study involving this polymorphism showed negative results accompanied by affective disorders 40. Moreover, Ching‐Lopes *et al*. (2015) found that the polymorphism of the corticotropin‐releasing hormone receptor 1 and 5‐hydroxytryptamine receptor 2A genes was also associated with depressive disorders 39. This emphasizes that other stages of the TRYCATs pathway are also important in the development of DD.

The next studied SNP was the c.803+221C>A polymorphism of TPH1 (rs18005832) localized at intron 7. The site is a potential GATA transcription factor‐binding site. The GATA transcription binding factors allow the initiation of transcription. Studies of the TPH1 polymorphism showed that the SNP affects expression of the gene. The genotypic and allelic distribution of the SNP was not associated with the occurrence of schizophrenia in Asian populations 41. However, we showed that the T/T genotype of the SNP may induce the development of DD in Polish patients. In addition, Jun *et al*. 42 revealed that the same SNP was also associated with the quality of life in women suffering from the irritable bowel syndrome.

In this paper, besides the SNP localized in *TPH1*, we also studied *TPH2* polymorphisms. 844G>T (rs4576025) was one of them. It may alter DNA–protein interactions, ultimately affecting transcription of the TPH2 gene, as the presence of the T allele is associated with a reduced TPH2 promoter activity 43, 44. As a consequence, serotonin synthesis is inhibited 45, 46. Moreover, studies showed that the A allele of the SNP was related to an increased risk of multiple sclerosis in the progressive subtypes of the disease among Finnish patients 47. The results of an earlier study showed that the TPH2 SNP was characteristic for depressed patients with suicidal attempts 48. The homozygous G allele (G/G genotype) frequency was higher in suicidal depressed patients when compared to control volunteers. Similarly, our study proved that the G/G genotype and allele G of c.‐844G>T*—TPH2* increased the risk of DD, whereas the G/T heterozygote and allele T of the same SNP were negatively correlated with the disease. However, a study on the Chinese Han population showed no association between SNP and depression. These discrepancies may result from demographic properties of the research groups—the difference in the number of women and men in the cited study 49. Furthermore, in another study on the Chinese population, the GG genotype of the same SNP carriers was more likely to enable depressive and anxiety symptom remission following escitalopram treatment compared with T allele carriers 50.

The last studied polymorphism presented in this paper is c.‐1449C>A (rs7963803) of *TPH2*. Yi *et al*. 51 found that the polymorphism was not correlated with occurrence of paranoid schizophrenia. Previous studies showed that two polymorphisms localized in the intron of *TPH2* (c.608+9108T>C—rs1386494 and rs1843809—c.608+5263G>T) were associated with major depression 52. During our experiment, we found a significant correlation between the studied TPH2 polymorphisms (rs4570625 and rs7963803) and the development of depression.

We are the first to investigate the association between combined genotypes of studied polymorphisms and DD. We also found a significant correlation between depressive disorders and haplotypes of the studied polymorphisms. Moreover, we were the first to demonstrate that three of six examined polymorphisms— *i.e*. rs1800532, rs10488682 and rs7963803—were associated with the risk of depression occurrence. The other three SNPs (previously examined by other researchers) were also found to strongly modulate DD development. For example, the AA genotype of c.‐1668T>A (rs623580) doubled the risk of DD (*P < *0.001). In turn, the G/G genotype and G allele increased the risk of the disease in the Polish population by five times, while the G/T genotype and T allele decreased this risk fivefold. In summary, the c.804‐7C>A, c.‐1668T>A, c.803+221C>A and c.‐173A>T polymorphisms of the TPH1 gene and the c.‐1449C>A, c.‐844G>T polymorphism of the TPH2 gene may be associated with depression in the Polish population. At the end of our article, we indicated and recommended that the relationship between the genes of TRYCATs enzymes and DD should be investigated further.

## Conclusion

We confirmed that SNPs of the genes involved in tryptophan metabolism, particularly the TRYCATs pathway, may have an impact on the risk of depressive disorders occurrence. We demonstrated that every studied SNP may modulate the development of depression. Therefore, these gene polymorphisms may be considered independent markers of depression. Our study supports the hypothesis that the TRYCATs pathways may be involved in the development of depression.

## Supporting information


**Figure S1.** Distribution of single‐nucleotide polymorphisms of genes encoding TPH1 and TPH2 and the age of the first episode of depression. Horizontal lines denote the average, while whiskers show the S.D.
