# Corrigendum

**DOI:** 10.1111/jcmm.13868

**Published:** 2018-09-25

In Wigner et al,^1^ the authors of the paper missed to include an acknowledgement section for the funding received for this research and would like to rectify the article to include the acknowledgement below:

The authors wished to apologize for any misunderstanding or inconvenience this may have cause.
