# Supplementary figures and images for: Association between single nucleotide polymorphisms of TPH1 and TPH2 genes, and depressive disorders
Source: J Cell Mol Med. 2018 Jan 5;22(3):1778–91. doi: 10.1111/jcmm.13459 (PMC5824396; doi:10.1111/jcmm.13459)

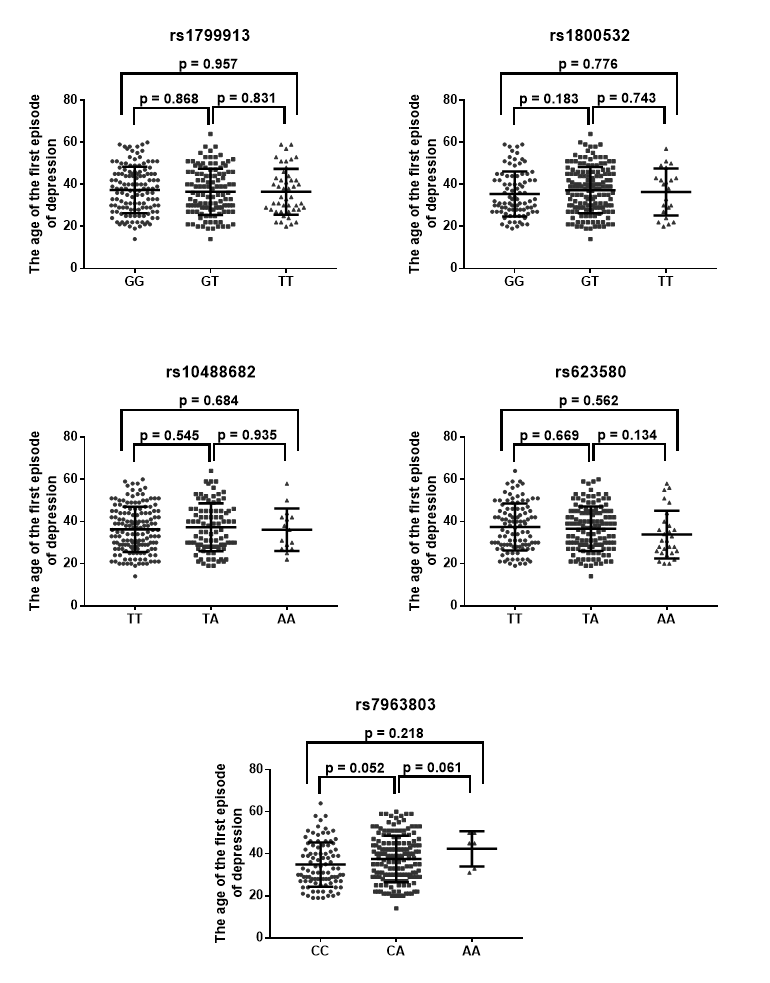

Supplement: Supplementary file 1 — Figure S1. Distribution of single‐nucleotide polymorphisms of genes encoding TPH1 and TPH2 and the age of the first episode of depression. Horizontal lines denote the average, while whiskers show the S.D. [file JCMM-22-1778-s001.tif]
